# Role of SCTR/AT1aR heteromer in mediating ANGII-induced aldosterone secretion

**DOI:** 10.1371/journal.pone.0222005

**Published:** 2019-09-03

**Authors:** Juan Bai, Karthi Duraisamy, Sarah O. K. Mak, Ahmed Allam, Jamaan Ajarem, Zhang Li, Billy K. C. Chow

**Affiliations:** 1 School of Biological Sciences, University of Hong Kong, Hong Kong, China; 2 Department of Zoology, College of Science, King Saud University, Riyadh, KSA; 3 Department of Zoology, Faculty of Science, Beni-Suef University, Beni-Suef, Egypt; 4 GHM Institute of CNS Regeneration, Jinan University, Guangzhou, China; Max Delbruck Centrum fur Molekulare Medizin Berlin Buch, GERMANY

## Abstract

The involvement of secretin (SCT) and its receptor (SCTR) in angiotensin II (ANGII)-mediated osmoregulation by forming SCTR/ angiotensin II type 1 receptor (AT1R) heteromer is well established. In this study, we demonstrated that SCTR/AT1R complex can mediate ANGII-induced aldosterone secretion/release through potentiating calcium mobilization. Through IHC and cAMP studies, we showed the presence of functional SCTR and AT1R in the primary zona glomerulosa (ZG) cells of C57BL/6N (C57), and functional AT1R and non-functional SCTR in SCTR knockout (SCTR^-/-^) mice. Calcium mobilization studies revealed the important role of SCTR on ANGII-mediated calcium mobilization in adrenal gland. The fluo4-AM loaded primary adrenal ZG cells from the C57 mice displayed a dose-dependent increase in intracellular calcium influx ([Ca^2+^]_i_) when exposed to ANGII but not from the SCTR^-/-^ ZG cells. Synthetic SCTR transmembrane (TM) peptides STM-II/-IV were able to alter [Ca^2+^]_i_ in C57 mice, but not the mice with mutated STM-II/-IV (STM-IIm/IVm) peptides. Through enzyme immunoassay (EIA), we measured the aldosterone release from primary ZG cells of both C57 and SCTR^-/-^ mice by exposing them to ANGII (10nM). SCTR^-/-^ ZG cells showed impaired ANGII-induced aldosterone secretion compared to the C57 mice. TM peptide, STM-II hindered the aldosterone secretion in ZG cells of C57 mice. These findings support the involvement of SCTR/AT1R heterodimer complex in aldosterone secretion/release through [Ca^2+^]_i_.

## Introduction

Fluid homeostasis is a fundamental survival mechanism to all terrestrial mammals to defend continuous osmotic stress from the surroundings. It helps the body to maintain not only fluid but also electrolyte balance, particularly sodium ions. Renin-angiotensin-aldosterone system (RAAS) is the major regulatory pathway within sodium homeostasis in which aldosterone production and release is mainly activated by angiotensin II (ANGII) release in adrenal cortex [1,2]. Aldosterone acts on kidney tubule cells and regulates ion channels to enhance sodium reabsorption and potassium (K^+^) excretion by interacting with mineralocorticoid receptor (MR), thereby indirectly influencing water retention and blood pressure [3]. Abnormal aldosterone secretion usually associates with hypertension, contributing to cardiac fibrosis and congestive heart failure. Aldosterone biosynthesis not only occur solely in adrenal zona glomerulosa (ZG) cells, but also has been reported to occur locally in various organs outside the adrenals, such as brain, heart and kidneys [1,4]. It is predominantly triggered by ANGII binding on angiotensin II type 1 receptor (AT1R) in ZG cells [2] and hereby elevating aldosterone production by increasing the expression of enzyme aldosterone synthase [cytochrome P450, family 11, subfamily B, polypeptide 2 (CYP11B2)] and steroidogenic acute regulatory protein (StAR) [5,6]. In rodents, both of the AT1R subtypes, AT1aR and AT1bR are found in ZG cells [7–9] and control aldosterone secretion via phosphatidylinositol-Ca^2+^ pathway [10–13], and involve the low threshold T-type and store-operated calcium channels (SOCE) [2,14,15] to elevate calcium (Ca^2+^) influx in ZG cells upon ANGII binding. Elevated Ca^2+^ level then affects the downstream Ca^2+^ dependent transcription factors—nuclear steroid receptor subgroup B (NGFI-B) and activator protein-1 (AP-1), which leads to the activation of CYP11B2 gene transcription, and, in turn, leads to aldosterone production [16–18].

In recent decades, it has been established that secretin (SCT) exerts an important modulatory function in ANGII-mediated osmoregulatory actions. SCT^-/-^ and SCTR^-/-^ mice models demonstrated that an intact SCT/SCTR axis is indispensable in mediating the central actions of ANGII in stimulating water-drinking behavior [19]. With this close association between SCT and ANGII in osmoregulation and the raising doctrine of G-protein coupled receptor (GPCR) dimerization, the synergistic relationship between SCT and ANGII can be possibly explained by the SCTR/AT1R heteromer formation. The existence of SCTR/AT1aR heteromer has been fully demonstrated *in vitro* by bioluminescence resonance energy transfer (BRET) assay. In addition, the interactions of SCTR/AT1aR heteromer can be inhibited by transmembrane (TM) peptides corresponding to the second and fourth TM segments of SCTR as well as the first and fourth TM segments of AT1aR [20]. Intracerebroventricular (i.c.v.) injections of these TM peptides, but not their mutant controls, were also able to abolish hyperosmolality-induced water drinking behavior [20] and attenuate ANGII/SCT-induced vasopressin release within hypothalamus [21]. All these evidence suggest that SCTR/AT1aR heteromer can mediate physiological function of ANGII in water homeostasis. On the other hand, we recently revealed that the absence of SCTR can result in a disrupted systemic RAAS and disrupt ANGII-induced aldosterone production in animal models [22]. Therefore, in this study, we tested our hypothesis that SCTR/AT1aR interactions can alter intracellular Ca^2+^ ([Ca^2+^]_i_) signaling to regulate aldosterone production. By monitoring *in vitro* [Ca^2+^]_i_ changes in a real-time manner upon ANGII stimulation, we investigated the effect of SCTR/AT1aR heteromer formation on calcium influx and consequent aldosterone production.

## Materials and methods

### Animals

All animal studies were approved by the Committee on the Use of Live Animals in Teaching and Research of the University of Hong Kong. The wild type C57BL/6N mice were bred and provided by the Laboratory Animal Unit (LAU), an AAALAC International accredited service unit of the Faculty of Medicine of the University of Hong Kong. The generation and genotyping of knockout mice for SCTR (SCTR^-/-^) has been described previously [23]. C57BL/6N (wild type, C57) and SCTR^-/-^ (N10) male mice aged 8 to 12 weeks with body weight 20 to 25 g were employed for all experiments. All animals were housed in a temperature-controlled room with a 12 h light-dark cycle and given free access to standard rodent chow and drinking water.

### Preparation of primary ZG cells

To prepare the primary ZG cells, respective mice were sacrificed by carbon dioxide euthanasia. The preparation of adrenal primary cells was based on description by Chu *et al*. [24]. Adrenal glands were immediately collected from euthanasic mice. The adrenocortical outer layer was carefully micro-dissected and digested in 10 ml Hank’s balanced salt solution (HBSS) (Gibco, Grand Island, NY, USA) with 0.01 g collagenase type II (Gibco), 0.01 g hyaluronidase (Sigma, St Louis, MO, USA) and 0.001 g pronase (Roche Diagnosis, Indianapolis, IN, USA) at 37°C, ambient air for 1 h. Tissue debris was then removed by a 100-μm cell strainer and the dispersed cells were obtained by centrifugation at 3,000 rpm, room temperature for 15 min. The cell pellet was washed twice with pre-warmed F-12K nutrient mixture (Gibco) containing 15% heat-inactivated horse serum (Invitrogen, Carlsbad, CA, USA), 2.5% heat-inactivated FBS (Invitrogen), 100 U/ml penicillin (Invitrogen), 100 μg/ml streptomycin (Invitrogen) and 250 ng/ml amphotericin B (Sigma). The cells were seeded in either six-well plates (Costar^®^, Corning, NY, USA) or 35 mm poly-D-lysine coated glass bottom petri dishes (MatTek, Ashland, MA, USA). For aldosterone release detection, cells with density at 10^6^ cells/ml, viability no less than 90% were seeded into the six-well plates with/-out ANGII (10 nM) and/or candesartan cilexetil (from 1 nM to 1 μM, antagonist of AT1Rs, Sigma) and/or 20 μg/ml synthetic TM peptides as specified (Table 1) for 0 min to 4 h. To monitor intracellular calcium oscillation, the seeding density was less than 10^4^ cells/ml to ensure the single cell layer. The cells were incubated at 37°C with 5% CO_2_-95% O_2._

**Table 1:** Sequences of C57 and mutated synthetic TM peptides used in this study.

Peptide^ʘ^	Sequence*
STM-I	KKMYTVGYSSSLAMLLVALSILCSFKK
STM-II	KKIHMHLFVSFILRALSNFIKDAVLKK
STM-IIm	KKIHMHLF**A**SFI**A**RA**A**SNF**A**KDAVLKK
STM-IV	LQAFVLFGWGSPAIFVALWAVTRHFLE
STM-IVm	LQAFVL**A**GW**A**SPA**A**FVALWA**A**TRHFLE

^ʘ^: Peptides were purchased from AnaSpec Inc. (Fremont, CA, USA), Phoenix Pharmaceuticals, Inc. (Burlingame, CA, USA) and GenScript USA Inc. (Piscataway Township, NJ, USA).

*: According to Lee *et al*. [20].

### Enzyme immunoassay (EIA)

The primary cultural media was obtained after removal of cells by centrifugation. Aldosterone concentration was then measured by enzyme immunoassays (EIA) using the aldosterone EIA kit (Enzo Life Sciences, Farmingdale, NY, USA) according to the manufacturer’s instructions.

### Immunohistochemical (IHC) staining

The adrenal glands were fixed in formalin and embedded in paraffin before three micron thick sections were prepared. IHC staining was performed as described by Lee *et al*. [19]. Briefly, sections were dewaxed using xylene before rehydrated in graded ethanol. Endogenous peroxidase activity was blocked with 5% hydrogen peroxide. Non-specific binding was prevented by incubating the sections in locked by 5% PBS supplemented with 5% (v/v) normal goat serum for 1 h at room temperature. SCTR antibody (1:250) was generated by Abmart (Shanghai, China). Immunostaining was visualized using HRP labeled secondary antibody available in SuperPicTure Polymer Detection Kit (Invitrogen). Sections from different animals were employed (N = 5) and imaged using a Nikon Eclipse 80i (Nikon, Tokyo, Japan).

### Real-time quantitative reverse transcription PCR (real-time qRT-PCR)

Total RNA was extracted from the adrenal glands with TRIzol reagent (Invitrogen). First-strand cDNAs were obtained (Transcriptor First Strand cDNA Secretion Kit, Roche Diagnostics GmbH, Mannheim, Germany), followed by quantitative PCR (7300 Real-Time PCR System, Applied Biosystems, Forster City, CA, USA). Specific Taqman probes (GAPDH: 4352339E, AT1aR: Mm00616371_ml, AT1bR: Mm02620758_S1, AT2R: Mm01341373_ml, Applied Biosystems) were used as specified by the manufacturer. 2^-ΔΔCt^ method [25] was employed for data analysis with the internal control, the gene of glyceraldehyde-3-phosphate dehydrogenase (*gapdh*).

### Tissue-based cAMP assay

To manifest the presence of functional SCTR in mouse adrenal gland, the cAMP level in response to SCT in the adrenal gland were determined using the LANCE^®^ cAMP 384 Kit (PerkinElmer Inc., Waltham MA, USA). Briefly, the whole adrenal gland from euthanasic mice was immediately collected and washed with ice-cold HBSS, before exposure to freshly prepared HBSS with/-out SCT (1 μM, AnaSpec, Fremont, CA, USA), at 37°C for 45 min. The glands were then homogenized in stimulation buffer freshly prepared as specified by the manufacturer and the protein concentration was determined. 10 μg total protein was then used for cAMP measurements. The signals were detected in Victor X4 (PerkinElmer).

### Intracellular calcium monitoring

Fluo4-AM (Invitrogen) was employed to label the free intracellular calcium ([Ca^2+^]_i_) as specified by the manufacturer. Briefly, the primary adrenal cortical cells were seeded in 35 mm glass-bottom petri dishes and recovered in F-12K media modified for primary ZG cells (around 12 h). After removing the media, the cells were washed with pre-warmed loading solution (HBSS-BSA, pH = 7.2, containing 25 mM HEPES (Sigma) and 0.1% W/V BSA (Roche Diagnosis, Indianapolis, IN, USA) in HBSS, with 2.5 mM probenecid (Invitrogen)), followed by incubation with loading solution freshly mixed with 1 μM Fluo4-AM and 0.02% Pluronic-127 (W/V, Invirogen) for 1 h at 37°C with 5% CO_2_-95% O_2_. The cells were then washed twice and kept in loading solution. All the loaded cells would be used within 3 h to ensure the viability. During the processing of live cells imaging, the unused cells were kept at 37°C with 5% CO_2_-95% O_2_. The Fluo4-AM loaded cells were then examined by the confocal laser scanning microscope (LSM 710 NLO, Carl Zeiss, Oberkochen, Germany). The ZG cells possessed large portion of nuclei, vesicles and round boundaries [26]. Only the attached single cells with diameter around 5–10 μm were used. The fluorescent intensity change was recorded via the time series recording with images captured every 2 sec. The fold change of fluorescent intensity was calculated according to the Eq 1. The mean intensity from the first minute was calculated as the baseline (F_o_). ANGII (0.1 nM, 10 nM and 1 mM, respectively, Phoenix Pharmaceuticals, Burlingame, CA, USA), KCl (60 mM, Sigma) and EGTA (5 mM, Sigma), respectively, was loaded at the time 0 or as specified time points. To study the effects of TM peptides, the cells were pre-incubated with corresponding synthetic peptides (Table 1) or candesartan (1 μM) at 37°C for 10 min before the imaging.

$$△F=\frac{F-Fo}{Fo}$$

**Equation 1. Calculation of fluorescent intensity fold change of primary adrenal ZG cells.** F: the actual fluorescent intensity of the cell at a specified time point. F_o_: the mean intensity in the first minute. ΔF: the fold change of fluorescent intensity of the cell at a specified time point. A time series recording the fluorescent intensity change was presented in a graph plotted according to the ΔF throughout the processing.

### Statistical analysis

All data are presented as means ± SEM, with the level of significance set at less than or equal to 0.05. The deviations between groups were analyzed using Prism 5.0 software (GraphPad Software Inc., San Diego, CA, USA). Unpaired t test was performed when 2 groups were under consideration, whereas data from more than 2 groups were analyzed by 1-way ANOVA, followed by Dunnett’s test.

## Results

### Presence of functional SCTR and AT1R in mouse ZG cells

**Fig 1:**
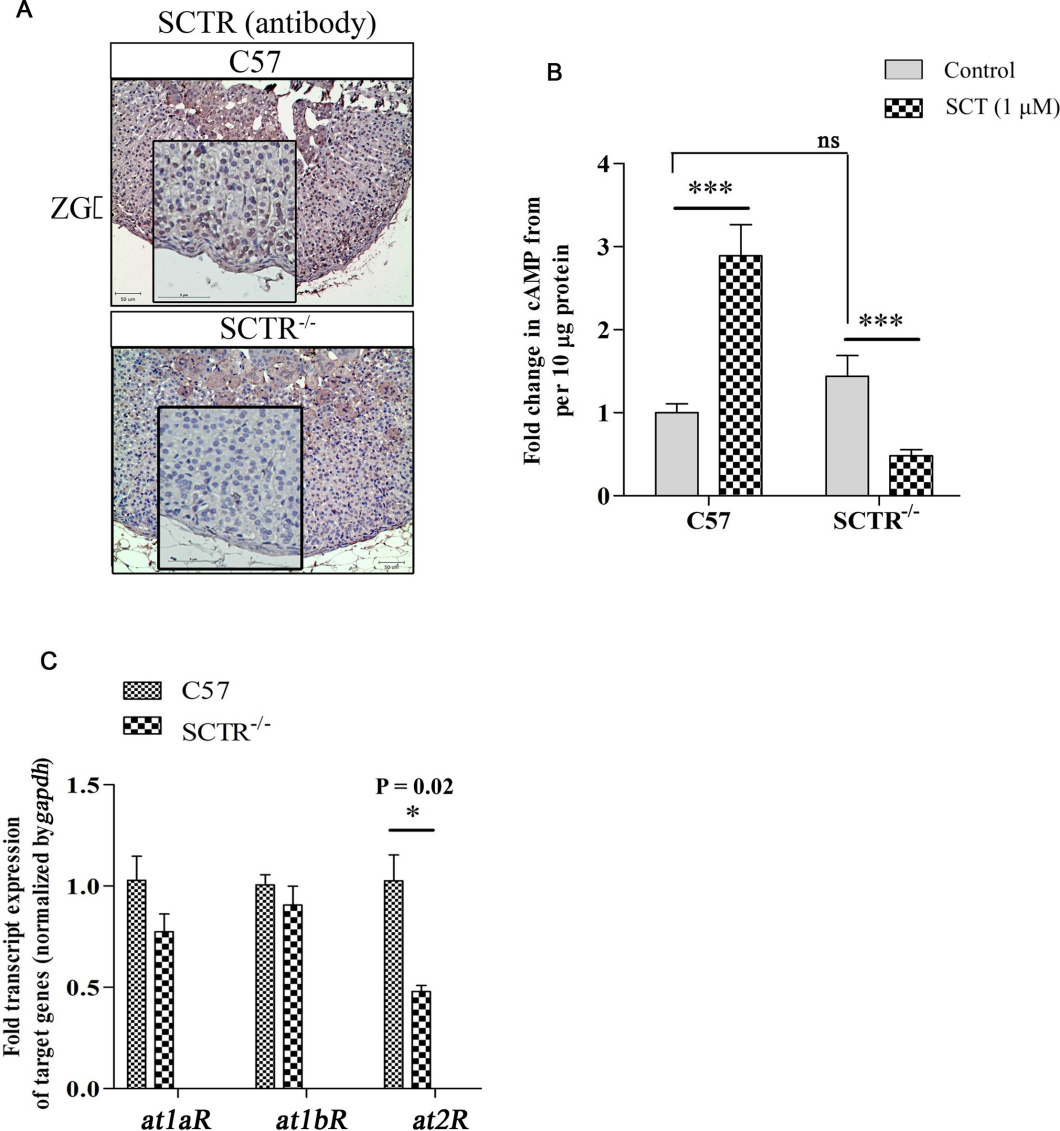
Functional SCTR and AT1R are present in mouse ZG cells. **(A)** The presence of SCTR in adrenal ZG cells. Representative images of immunohistochemical staining of sections obtained from at least 5 independent animals for each genotype and they were captured from at least 3 stained sections of every independent animal. Sections from SCTR^-/-^ were employed as a negative control for SCTR staining. Scale bar represents 50 μm in outer panel and 5 μm in the zoomed fields. **(B)** cAMP assays showing the presence of functional SCTR in mouse adrenal gland. ***, P < 0.001 *vs* control group in respective genotype. Data are presented as the mean + SEM of at least three independent experiments performed in duplicates. **(C)** Transcripts of *AT1aR*, *AT1bR* and *AT2R* in ZG tissue determined by Taq-man probes normalized with internal control gene *gapdh*. *, P < 0.05 *vs* corresponding control in C57 mice. N = 4–5. ***, P < 0.001; *, P < 0.05 *vs* DMSO group. Data are presented as the mean + SEM of four independent experiments performed at least in duplicates.

To investigate the receptor heteromer functionality in aldosterone secretion, we first studied the presence of functional SCTR and ANGII receptors in the ZG layer. By IHC staining, we showed the presence of SCTR in ZG cell layer of C57 mice, but not in ZG cell layer of SCTR^-/-^ mice (Fig 1A). Meanwhile, SCT (1 μM) was able to stimulate cAMP production in adrenal preparations from C57 mice, but not in SCTR^-/-^ mice (Fig 1B), indicating the presence of functional SCTR in mouse adrenal gland. For the presence of AT1R in adrenal ZG cells, all three ANGII receptors: AT1aR, AT1bR and AT2R have been reported to be present in rat adrenal ZG [27]. In this report, we showed the transcripts level for *AT1aR*, *AT1bR* and *AT2R* from dissected ZG tissues of C57 mice. There were no detectable differences in the transcript levels for *AT1aR* and *AT1bR* in C57 and SCTR^-/-^ mice. However, there is a significant reduction in the expression of *AT2R* mRNA in SCTR^-/-^ ZG tissues when compared to C57 mice (Fig 1C). To show the presence of functional AT1Rs in ZG cells, we next used the antagonist of AT1Rs, candesartan to incubate with ZG primary cell culture and observed the effect in ANGII-induced aldosterone release. In summary, we showed the presence and absence of functional SCTR in C57 and SCTR^-/-^ mouse ZG cells respectively. Both C57 and SCTR^-/-^ mice possess functional AT1Rs.

### Effect of SCTR on ANGII-mediated calcium mobilization

**Fig 2:**
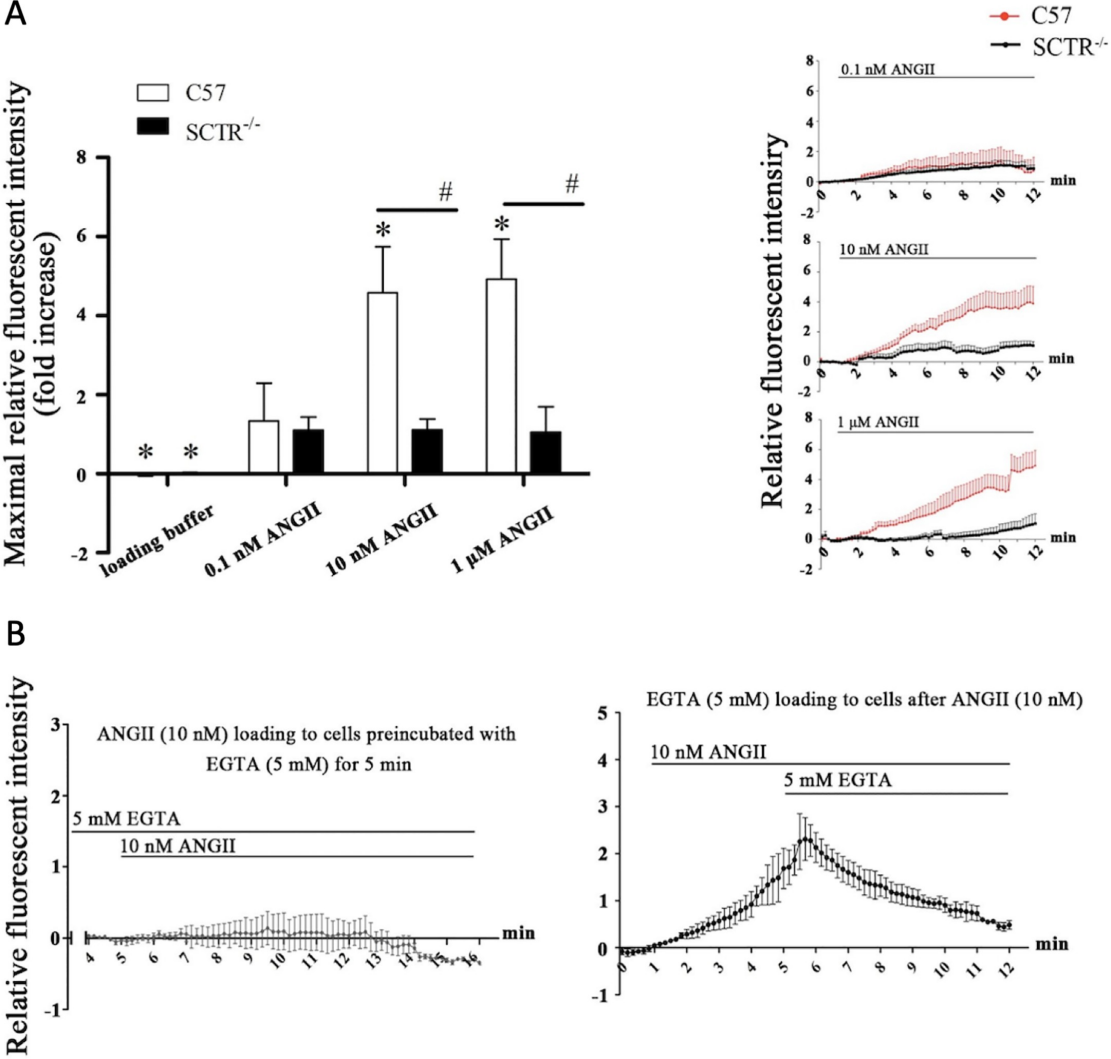
SCTR potentiates ANGII-induced increase in [Ca^2+^]_i_. **(A)** [Ca^2+^]_i_ change in primary ZG cells obtained from C57 and SCTR^-/-^ mice, in response to ANGII (ranging from 0.1 nM to 1 μM). ANGII dose-dependently increased [Ca^2+^]_i_ in C57 ZG cells. The tested ANGII dosages lead to a minor increase in [Ca^2+^]_i_ in SCTR^-/-^, similar to the 0.1 nM ANGII-treated ZG cells of C57 mice. The maximal ANGII-induced [Ca^2+^]_i_ increase was recorded at around 9 min, 11 min and 12 min post-ANGII loading, depending on the dosage of ANGII. Loading buffer was loaded as negative control. *, P <0.05 *vs* 0.1 nM ANGII-treatment in corresponding genotype. ^#^, P < 0.05, *vs* counterparts in C57 mice. Data are present as mean + SEM from at least five independent experiments. **(B)** Extracellular calcium is a prerequisite for ANGII-induced Ca^2+^ mobilization and is the major source of [Ca^2+^]_i_ influx. Pre-exposure to EGTA (5 mM) prior to ANGII (10 nM) abolished extracellular Ca^2+^ mobilization in C57 ZG cells. In contrary, increased [Ca^2+^]_i_ upon ANGII was observed, until EGTA was loaded. The [Ca^2+^]_i_ influx induced by ANGII was gradually and eventually chelated by EGTA nearly back to the resting level. Data are present as mean ± SEM from three independent experiments.

It has already been shown that the calcium-CaMKs pathway is responsible for mediating ANGII’s regulatory effects on aldosterone secretion [2]. Our finding is consistent with this observation, as the C57 primary ZG cells exhibited increased [Ca^2+^]_i_ upon ANGII stimulation in a dose dependent manner (0.1 nM to 1 μM) (Fig 2A). However, this response was absent in ZG cells prepared from SCTR^-/-^ mice (Fig 2A), which evidently indicated the involvement of SCTR in the ANGII-[Ca^2+^]_i_ pathway. As there is calcium mobilization in ZG cells, in order to determine the source of Ca^2+^, we used EGTA to chelate the extracellular Ca^2+^ ions. C57 ZG cells pre-incubated with 5 mM EGTA were non-responsive to 10 nM ANGII stimulation as EGTA chelates the extracellular calcium ions (Fig 2B). Moreover, administration of EGTA 5 mins after 10 nM ANGII stimulation was able to reduce [Ca^2+^]_i,_ levels immediately, which clearly indicates that ANGII-induced Ca^2+^ influx was originated from extracellular source (Fig 2B).

### Effects of synthetic TM peptides on ANGII-mediated calcium mobilization

**Fig 3:**
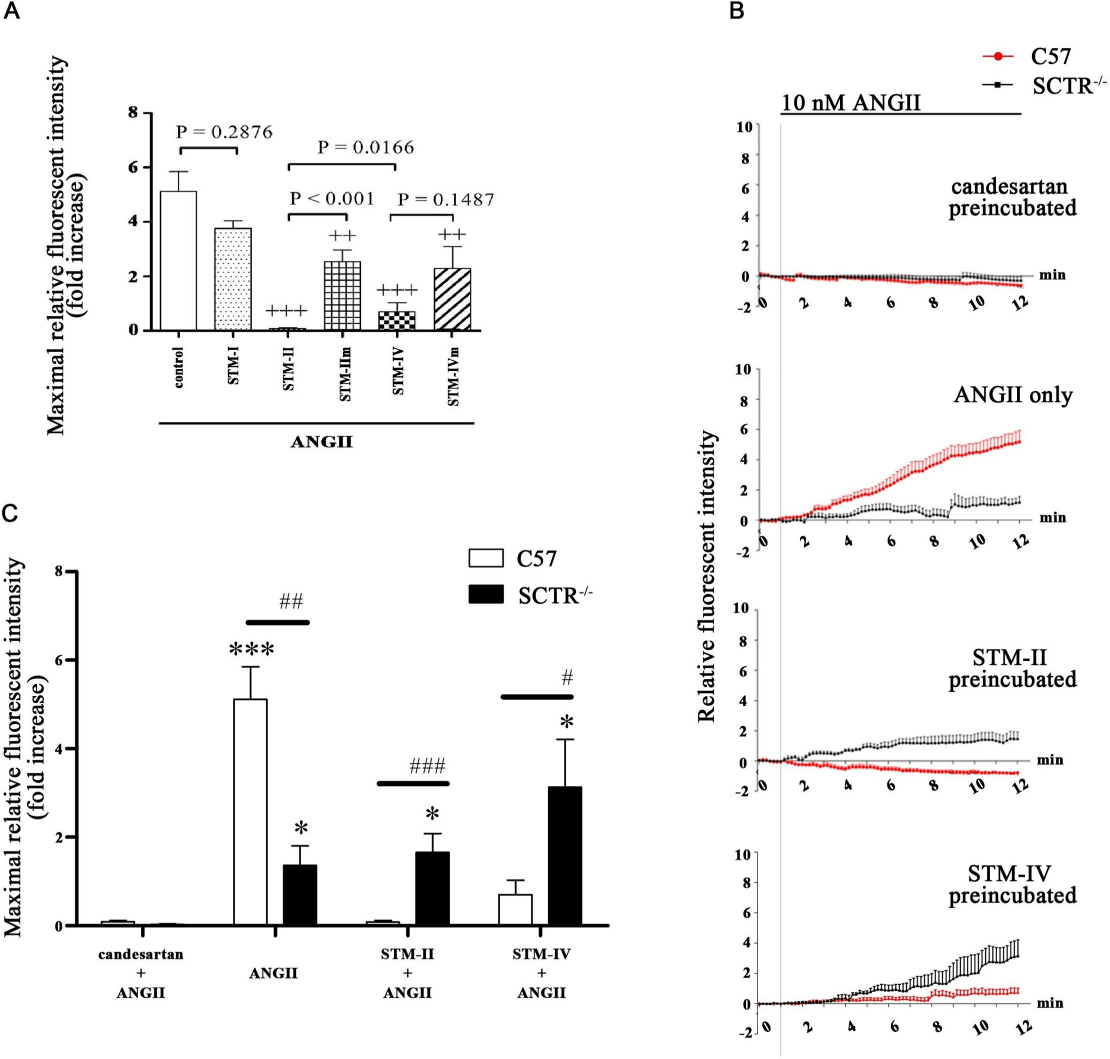
SCTR/AT1R hetero-complex mediates ANGII-induced [Ca^2+^]_i_ in C57 mice. **(A)** STM-II/-IV, but not their mutants, inhibited ANGII-induced [Ca^2+^]_i_. Non-dimer-involved peptide STM-I had no effect on the ANGII-induced [Ca^2+^]_i_. **(B)** and **(C)** The real-time records of [Ca^2+^]_i_ and the maximal [Ca^2+^]_i_, respectively, in ZG cells from C57 and SCTR^-/-^ in response to ANGII (10 nM) with the presence or absence of candesartan (1 μM) or STM peptides (20 μg/ml). The primary ZG cells were pre-incubated with/-out candesartan or STM-II/-IV for 10 min before ANGII loading. In C57 mice, compared to ANGII-only treatment, the presence of STM-II/-IV abolished the [Ca^2+^]_i_ increase like candesartan. This inhibitory effect of STM-II/-IV was absent in SCTR^-/-^. Candesartan prevented ANGII-induced [Ca^2+^]_i_ elevation in both C57 and SCTR^-/-^ mice. STM-IIm/-IVm showed much less pronounced inhibitory effects than STM-II/-IV in C57 mice. ^+++^, P < 0.001; ^++^, P < 0.01 *vs* ANGII treatment. ***, P < 0.001; *, P < 0.05 *vs* candesartan-pretreated group in corresponding genotype. ^###^, P < 0.001; ^##^, P < 0.01; ^#^, P < 0.05 *vs* C57 mice in respective treatment. Data are present as mean + SEM from at least five independent experiments.

Comprehensive data provided from our previous work have shown that certain synthetic TM peptides synthesized from the specific TM segments of SCTR or AT1aR are able to disrupt SCTR homomer, AT1aR homomer and/or SCTR/AT1aR heteromer formation in *in vitro* studies [20]. Moreover, these TM peptides were able to inhibit hyperosmolality-induced water intake and alter ANGII/SCT-induced vasopressin release when injected into the lateral ventricle in C57 mice [20,21]. In this study, we utilized STM-II, which disrupts the formation of SCTR/AT1aR heteromer, and STM-IV which disrupts the SCTR homomer and SCTR/AT1aR heteromer formation to investigate the possibility of SCTR/AT1aR heteromer in mediating ANGII-aldosterone production. Respective mutant peptides STM-IIm and STM-IVm were used as control to investigate the function of SCTR/AT1R complex on ZG cell surface. We found that STM-II/-IV, but not STM-IIm/-IVm, were able to abolish ANGII-induced [Ca^2+^]_i_ in C57 ZG cells (Fig 3A). Similar to C57 mice, ANGII-induced [Ca^2+^]_i_ in SCTR^-/-^ mice was completely abolished by a known AT1Rs antagonist, candesartan (1 μM) (Fig 3B and 3C). While, STM-II and STM-IV also displayed similar inhibitory effects as candesartan but they were not able to completely abolish the basal Ca^2+^ levels in SCTR^-/-^ ZG cells. STM-II and STM-IV were only able to partially hinder the downstream effect whereas candesartan completely antagonized AT1R function. These results collectively showed that AT1R is responsible for mediating the ANGII-induced [Ca^2+^]_i_ via SCTR/AT1aR heteromer. Specificity of TM peptides were also tested using a non-specific TM peptide, STM-I (Fig 3A) that is known to have no effect on the disruption of receptor homomer or heteromer [20].

### Effect of SCTR on ANGII-induced aldosterone release

**Fig 4:**
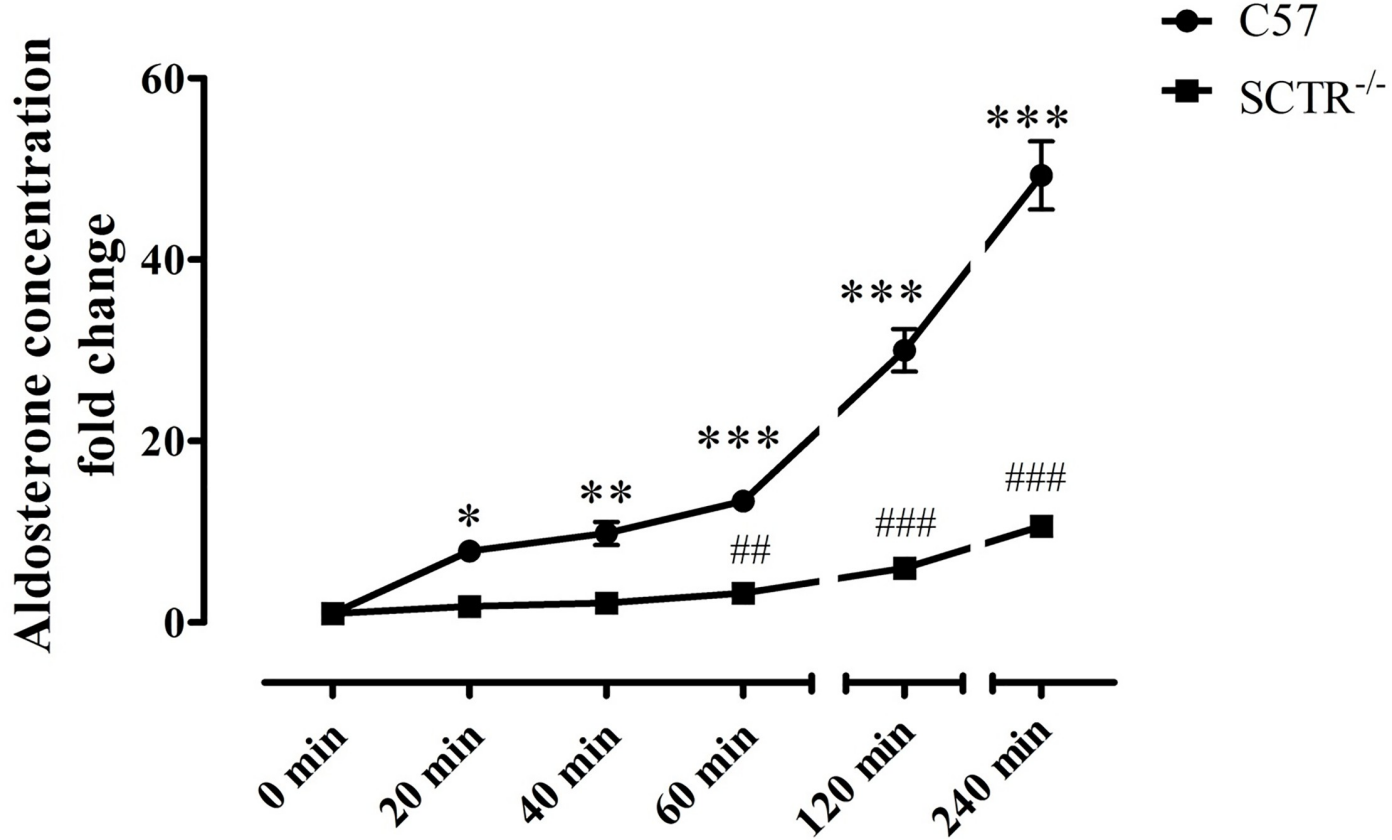
SCTR potentiates ANGII-induced aldosterone release. The dispersed primary ZG cells obtained from C57 and SCTR^-/-^ were exposed to ANGII (10 nM). A significant increase in aldosterone release was generated by the presence of ANGII within 20 min in C57 mice, but not until 60 min in SCTR^-/-^ mice in tested time points. This stimulatory effects lasted for at least four hours as observed. ***, P < 0.001; **, P < 0.01; *, P < 0.05 *vs* start point in C57. ^###^, P < 0.001; ^##^, P < 0.05 *vs* 0 min in SCTR^-/-^. N = 4–5. Data are presented as the mean ± SEM.

To investigate the effect of SCTR in ANGII-induced (10 nM) aldosterone release, we measured cumulative aldosterone release from dispersed ZG cells prepared from C57 and SCTR^-/-^ mice. As early as 20 mins after ANGII stimulation, aldosterone levels were significantly elevated in the culture medium of C57 ZG primary cells, and this stimulatory action of ANGII lasted for at least for 4 h. In SCTR^-/-^ ZG cells, the stimulatory action of ANGII was significantly reduced compared to the C57 ZG cells (Fig 4), and these data are consistent with our earlier *in vivo* observations that ANGII-induced aldosterone production requires an intact SCT-SCTR axis [22].

### Effect of STM-II on ANGII-induced aldosterone release

**Fig 5:**
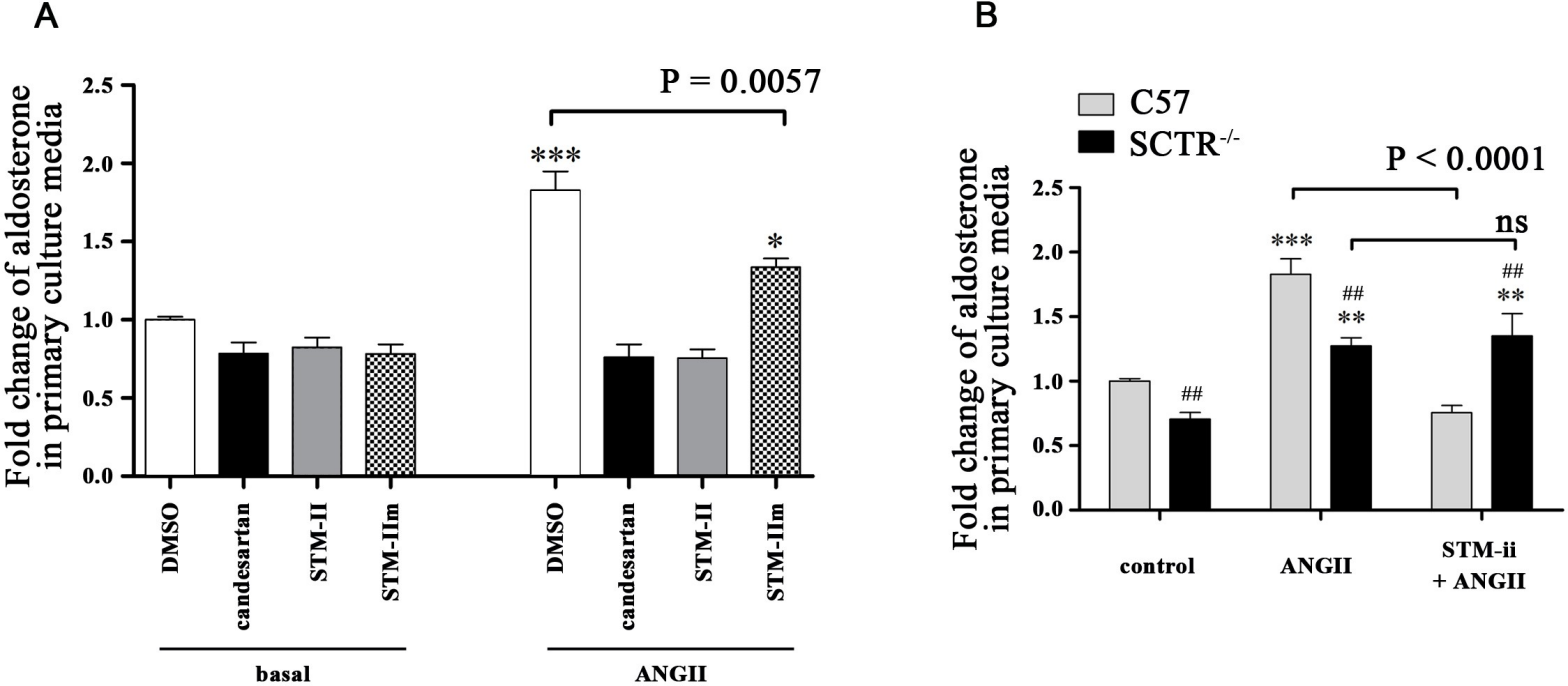
SCTR/AT1R hetero-complex is responsible for ANGII-induced aldosterone release in C57 mice. Primary ZG cells prepared from C57 or SCTR^-/-^ mice were pre-incubated with candesartan (1 μM), STM-II (20 μg/ml) or STM-IIm (20 μg/ml) for 30 min, respectively, before co-incubation with ANGII (10 nM) for another 1 h. **(A)** STM-II, but not STM-IIm, prevented ANGII-induced aldosterone secretion in primary C57 ZG cells, similar to the effects of candesartan. **(B)** The inhibitory effects of STM-II on the ANGII-induced aldosterone release was absent in SCTR^-/-^. With the presence or absence of STM-II, SCTR^-/-^ secreted similar aldosterone levels upon ANGII, significantly higher than the control group. Besides, the 1 h aldosterone production in control SCTR^-/-^ ZG cells showed the attenuated intrinsic secretion. ***, P < 0.0001; **, P < 0.01; *, P < 0.05 *vs* respective treatment in basal group (A) or control group in corresponding genotype (B). ^##^, P < 0.01 *vs* C57 mice in respective treatment. N = 8–12. Data are present as mean + SEM.

As SCTR/AT1R heteromer is involved in ANGII-induced calcium mobilization, further experiments were performed to investigate the effect of SCTR/AT1R complex on subsequent aldosterone release in response to ANGII. Candesartan and STM-II or STM-IIm were used to measure the basal aldosterone release from ZG cells (Fig 5A). On the other hand, 1 h incubation with 10 nM ANGII can dramatically induced aldosterone release in ZG cells (Fig 5A). This stimulation was reduced to the basal level in the presence of STM-II or candesartan (Fig 5A). STM-IIm had a much smaller inhibitory effect when compared to STM-II, which is consistent with the pattern changes observed in [Ca^2+^]_i_ (Fig 3). In ZG cells from SCTR^-/-^ mice, ANGII-induced aldosterone release was much less pronounced, while incubation with STM-II has no effect on this release (Fig 5B). These data collectively suggest the formation of SCTR/AT1R heteromer is required for a well-pronounced ANGII-induced aldosterone release in C57 adrenal ZG cells.

## Discussion

ANGII initiates aldosterone production in ZG cells via sustained [Ca^2+^]_i_ through activating the low-voltage-gated Ca^2+^ channels on the plasma membrane, and triggering the storage release from endoplasmic reticulum (ER) [2,15]. This increased [Ca^2+^]_i_ is critical for the subsequent CaMKs-regulated transcription regulators controlling CYP11B2 expression [28,29]. From our finding, by correlating [Ca^2+^]_i_ changes and aldosterone release upon ANGII stimulation in ZG cells of C57 and SCTR^-/-^ mice, we found that functional SCTR is required for a well-pronounced function of ANGII in Ca^2+^ mobilization and aldosterone release. Addition of EGTA before and after the treatment of ANGII with ZG cells showed that the extracellular Ca^2+^ is the major source of [Ca^2+^]_i_ upon the activation. Therefore, the observed attenuated [Ca^2+^]_i_ and aldosterone secretion in SCTR^-/-^ is likely to be the result of a lowered activity of the low-voltage-gated Ca^2+^-channels.

Intriguingly, although SCTR/AT1R heterodimer is absent in the SCTR^-/-^ mice, ANGII-induced [Ca^2+^]_i_ is completely inhibited by the presence of candesartan. However, STM-II or STM-IV were only able to partially inhibit the [Ca^2+^]_i_, indicating that AT1R alone can mediate the ANGII-induced [Ca^2+^]_i_ in a heteromer-independent manner. The aldosterone production in SCTR^-/-^ ZG cells in response to ANGII treatment is significantly less than that of the C57 ZG cells, which indicates the disturbed ANGII-aldosterone axis without the functional SCTR that is also consistent with our earlier *in vivo* results [22]. Therefore, disturbance in functional heteromer results in reduction of [Ca^2+^]_i_, hence reduction in aldosterone secretion. On the other hand, we observed a reduced intrinsic *at2R* in the SCTR^-/-^ ZG cells. Since, AT2R is commonly known to counteract the effects of AT1R [30], it is likely that this reduced intrinsic AT2R can enhance the actions of ANGII-AT1R in SCTR^-/-^ mice, compensating for the absence of SCTR/AT1R heteromer, hence there is calcium release and aldosterone production in SCTR^-/-^ ZG cells.

In this study, we showed the functional AT1Rs and SCTR are present in the mouse adrenal gland. Unfortunately, at present, there are no known commercially available biochemical tools to distinguish the two different isoforms of type 1 receptor in rodent, AT1aR and AT1bR, which share high similarity in sequence, structure and functions [31]. However, from our previous work, we have demonstrated that SCTR is able to hetero-oligomerize with both of these receptors, while only the SCTR/AT1aR heteromer formation *in vitro* can be blocked by the use of transmembrane peptides STM-II, STM-IV, ATM-1 and ATM-4 [20]. Even though we have demonstrated the interaction of SCTR/AT1aR in heterologous cell lines using BRET assay, we acknowledge that there is still a lack of direct evidence of SCTR/AT1aR interaction in adrenal cells due to the limitation of the current study. However, from the data observed using TM peptides, it is evident that SCTR forms a complex with AT1aR and participates in aldosterone secretion. Of note, it is known that SCTR can form a heteromer with AT1bR. Meanwhile, we have also observed the expression level of *AT1bR* in ZG cells of both C57 and SCTR^-/-^ mice as similar to *AT1aR*, therefore we cannot neglect the possible functions of SCTR/AT1bR heteromer in adrenal gland. Nevertheless, in this study, STM-IIm/-IVm peptides also produced a much smaller inhibitory effect on [Ca^2+^]_i_ and aldosterone production in C57 mice, suggesting that even the mutated ones can hinder the formation of the SCTR/AT1aR complex to some extent. However, STM-II and STM-IV exhibited similar inhibitory effects as candesartan, a type 1 receptor antagonist of both AT1aR and AT1bR [32], on ANGII-induced Ca^2+^ mobilization. Particularly, STM-II dramatically inhibited Ca^2+^ mobilisation and aldosterone release in C57 mice. By taking all these data in consideration, it can be concluded that SCTR/AT1aR heteromer is necessary for mediating the calcium mobilization to induce aldosterone secretion.

## Supporting information

S1 Fig Less increased [Ca^2+^]_i_ in response to ANGII was observed in SCTR^-/-^ primary ZG cells. Shown are the real-time images representing Fluo4-AM labelled free [Ca^2+^]_i_ in primary ZG cells obtained from C57 and SCTR^-/-^ mice. ANGII (10 nM) was loaded at time 1 min. Fluorescent intensity in C57 mice was rapidly increased and lasted for minutes. While this increase was less pronounced in SCTR^-/-^. All fluorescent images were acquired under similar settings and were representative of at least five independent experiments. Scale bar represents 10 μm.
(TIFF)
