# Supplementary figures and images for: Role of SCTR/AT1aR heteromer in mediating ANGII-induced aldosterone secretion
Source: PLoS One. 2019 Sep 3;14(9):e0222005. doi: 10.1371/journal.pone.0222005 (PMC6719825; doi:10.1371/journal.pone.0222005)

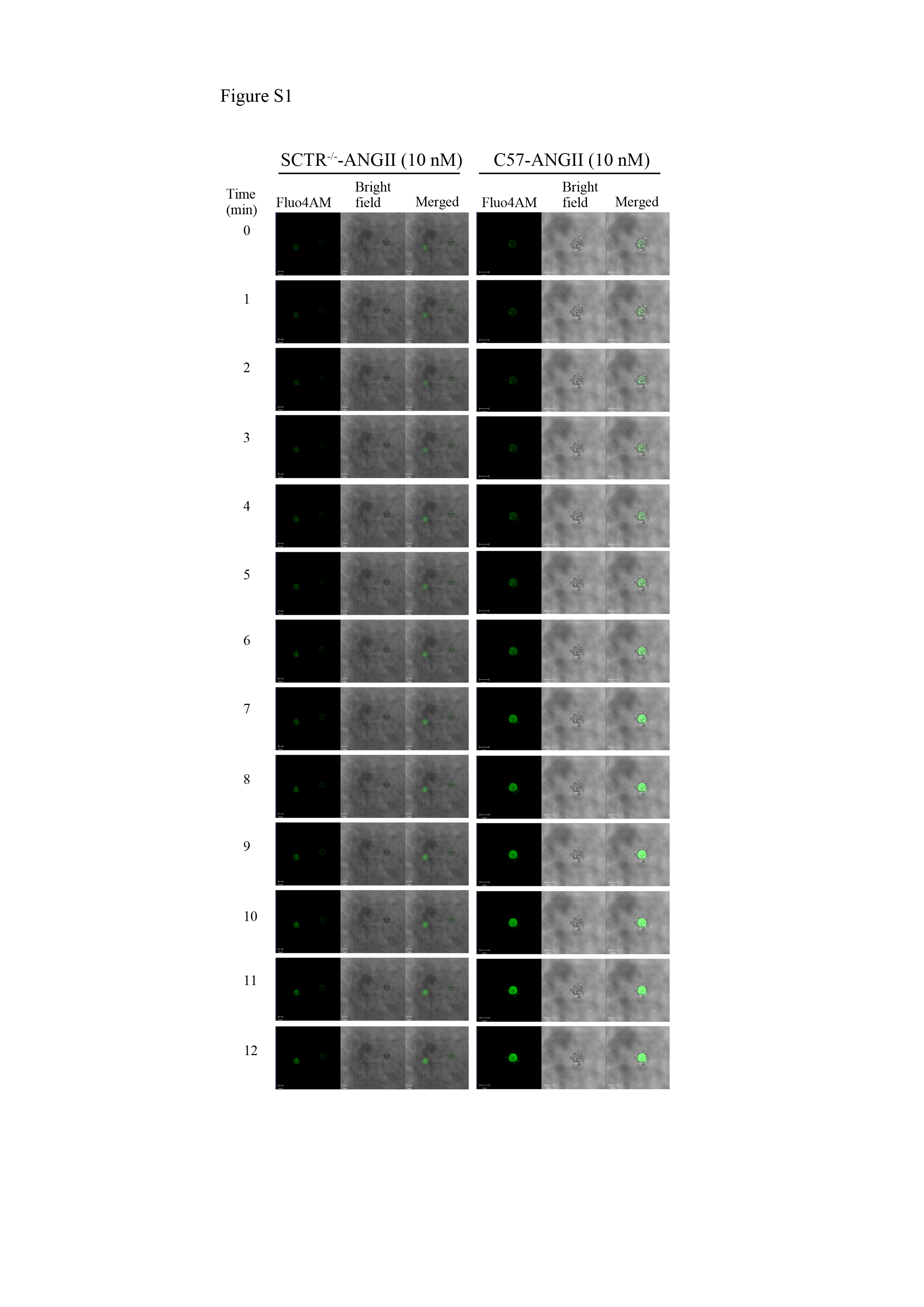

Supplement: S1 Fig — Shown are the real-time images representing Fluo4-AM labelled free [Ca2+]i in primary ZG cells obtained from C57 and SCTR-/- mice. ANGII (10 nM) was loaded at time 1 min. Fluorescent intensity in C57 mice was rapidly increased and lasted for minutes. While this increase was less pronounced in SCTR-/-. All fluorescent images were acquired under similar settings and were representative of at least five independent experiments. Scale bar represents 10 μm. (TIFF) [file pone.0222005.s001.tiff]
